# Manganese in residential drinking water from a community-initiated case study in Massachusetts

**DOI:** 10.1038/s41370-023-00563-9

**Published:** 2023-06-10

**Authors:** Alexa Friedman, Elena Boselli, Yelena Ogneva-Himmelberger, Wendy Heiger-Bernays, Paige Brochu, Mayah Burgess, Samantha Schildroth, Allegra Denehy, Timothy Downs, Ian Papautsky, Birgit Clauss Henn

**Affiliations:** 1 Department of Environmental Health, Boston University School of Public Health, Boston, MA, USA.; 2 Department of Biomedical Engineering, University of Illinois Chicago, Chicago, IL, USA.; 3 Department of International Development, Community, and Environment, Clark University, Worcester, MA, USA.; 4 Holliston Community Member, Holliston, MA, USA.

**Keywords:** Metals, Environmental monitoring, Children’s exposure/health

## Abstract

**BACKGROUND:**

Manganese (Mn) is a metal commonly found in drinking water, but the level that is safe for consumption is unknown. In the United States (U.S.), Mn is not regulated in drinking water and data on water Mn concentrations are temporally and spatially sparse.

**OBJECTIVE:**

Examine temporal and spatial variability of Mn concentrations in repeated tap water samples in a case study of Holliston, Massachusetts (MA), U.S., where drinking water is pumped from shallow aquifers that are vulnerable to Mn contamination.

**METHODS:**

We collected 79 residential tap water samples from 21 households between September 2018 and December 2019. Mn concentrations were measured using inductively coupled plasma mass spectrometry. We calculated descriptive statistics and percent of samples exceeding aesthetic (secondary maximum containment level; SMCL) and lifetime health advisory (LHA) guidelines of 50 μg/L and 300 μg/L, respectively. We compared these concentrations to concurrent and historic water Mn concentrations from publicly available data across MA.

**RESULTS:**

The median Mn concentration in Holliston residential tap water was 2.3 μg/L and levels were highly variable (range: 0.03–5,301.8 μg/L). Mn concentrations exceeded the SMCL and LHA in 14% and 12% of samples, respectively. Based on publicly available data across MA from 1994–2022, median Mn concentration was 17.0 μg/L (*N* = 37,210; range: 1–159,000 μg/L). On average 40% of samples each year exceeded the SMCL and 9% exceeded the LHA. Samples from publicly available data were not evenly distributed between MA towns or across sampling years.

## INTRODUCTION

Safe drinking water is fundamental for health. Yet, unregulated contaminants, like manganese (Mn), have been detected in public drinking water supplies in the United States (U.S.) and globally at levels that may pose a public health concern [1–3]. Mn is a metal that comprises approximately 0.1% of the earth’s crust, and is a component of soil and rock, resulting in the natural enrichment of Mn in drinking water sources [1]. Mn is also commonly used as a fungicide in agriculture, and as a key component in steel production, which results in agricultural run-off and industrial contamination of drinking water sources [4]. Further, communities that rely on very shallow aquifers ( < 50 feet to water table) as their main source of drinking water are particularly vulnerable to Mn [1, 5].

Drinking water concentrations of Mn are not federally regulated and Mn is classified by the Environmental Protection Agency (EPA) as a secondary drinking water contaminant in the U.S. The Secondary Maximum Contaminant Level (SMCL), set at 50 μg/L, is a non-enforceable guideline based on aesthetic concerns (e.g., color, taste) [6]. The EPA U.S. lifetime health advisory (LHA), set at 300 μg/L, is a non-enforceable guideline at which daily intake at this level or below for the general population is not thought to be associated with adverse health effects [7]. Under the Safe Drinking Water Act, the Unregulated Contaminant Monitoring Rule (UCMR) was developed to measure the occurrence of unregulated contaminants and determine if a primary (i.e., enforceable) standard should be considered [8]. While Mn has been federally monitored under the UCMR in some program years, no primary standards have been developed.

Based on national data from the U.S. Geological Survey (USGS), which sampled over 40,000 groundwater wells between 1988 and 2017, 31% of samples exceeded the SMCL of 50 μg/L and 13% exceeded the LHA of 300 μg/L for Mn. Authors noted the highest groundwater Mn concentrations were in northeastern U.S., including Massachusetts (MA), where 30% of samples exceeded the LHA [3]. Similarly, a recent study examined concentrations of Mn in drinking water samples taken as part of the UCMR third (2012–2015) and fourth (2018–2020) programs and reported that between 13 and 17% of public water systems (PWS) had a sample exceeding 50 μg/L and between 2 and 3% had a sample exceeding 300 μg/L [9]. Authors noted that Mn concentrations in drinking water were higher in groundwater samples compared to surface water samples and, consistent with the USGS report, the highest water Mn concentrations were observed in northeastern U.S [3, 9]. These are among the few studies that have examined Mn in drinking water in the U.S., and they were limited to a small number of sampling locations over a narrow range of sampling years.

The level of Mn in drinking water that is considered safe, particularly for vulnerable subpopulations like children, remains unknown. Mn is both an essential nutrient and established neurotoxicant [4, 10], whereby deficient and excess levels of Mn have been associated with neurotoxicity [11–14]. In adults, one study reported an association between exposure to high concentrations of Mn in water with adverse neurological outcomes, including Parkinson-like symptoms [15]. Children, compared to adults, are more vulnerable to excess Mn exposure given the increased demand for Mn to support growth and development, as well as higher intakes per unit body weight and additional exposure sources (e.g., formula or infant foods) [4, 16]. Children may also be more susceptible than adults to the adverse effects of Mn, given their underdeveloped homeostatic mechanisms and the complex dynamics underlying brain development in early life [11, 17]. Increasing evidence links Mn in drinking water to decrements in cognitive function and other neurobehavioral outcomes in children [2]. In studies worldwide that have reported decrements in neurobehavior, water Mn concentrations were as high as 8600 μg/L [18–21], which is within the range of Mn concentrations measured in USGS water samples [3]. However, adverse associations with neurobehavioral outcomes among school-aged children, such as lower IQ scores, decrements in intellectual function, and decreased learning and memory scores [22, 23], have been reported in studies with much lower average water Mn levels, including concentrations that are below current U.S. guidelines (i.e., SMCL of 50 μg/L and LHA of 300 μg/L) [18]. While evidence suggests that Mn in drinking water may pose a threat to children’s health, there remains a paucity of data on individual-level residential exposure to Mn in drinking water in the U.S. that adequately characterize temporal and spatial variations in water Mn concentrations.

Our case study examines Mn concentrations from residential tap water samples collected in a suburban community in MA that relies on very shallow aquifers for nearly 100% of their drinking water [5, 24]. As our previous work has demonstrated, this aquifer system contains naturally high concentrations of Mn and has been, and continues to be, impacted by existing and legacy sources such as landfills, industry, and contamination events [5]. This pilot study was motivated by community members who reported experiencing episodic discolored drinking water for years, and who continue to be partners in the ongoing research [5, 24]. We measured Mn concentrations in repeated residential tap water samples, explored temporal and spatial variability in water Mn concentrations, and compared concentrations with publicly available statewide data.

## METHODS

### Holliston, MA case study

Data were collected as part of the ACHIEVE (Assessing Children’s Environmental Exposures) study, a community-initiated pilot research study in Holliston, MA. Holliston is a bedroom community of the Boston metropolitan area with a population of about 15,000 [25]. The ACHIEVE study was designed in response to community concerns about drinking water quality and their children’s health [24]. The primary objectives of the pilot study were to assess children’s exposure to environmental contaminants during critical periods of development using naturally shed baby teeth, and to foster a collaboration with community members to implement the study and communicate findings. Details about study design and procedures have been published elsewhere [24]. Briefly, mother-child pairs were eligible to participate if they: a) were residents of Holliston, MA; b) had a child aged 5–13 years at time of enrollment who had lost or was losing teeth; c) lived in Holliston during pregnancy with participating child; and d) were willing to donate their child’s shed tooth.

Thirty mother-child pairs were enrolled in the pilot study between 2017 and 2018. Questionnaires, administered to mothers at enrollment, collected information on participant sociodemographic characteristics, current drinking water consumption patterns, use of filters for drinking water, parent-reported learning and behavioral disorders in participating children, and beliefs about water quality and their children’s health. In 2018, as a second phase of the ACHIEVE study, participants were invited to participate in residential tap water sampling conducted between 2018 and 2019. A subset (*n* = 21, 72%) of the original 30 participants volunteered and were enrolled in drinking water sampling, which comprises the analytical sample for the present case study. Participants provided written informed consent prior to participation. The research study protocol and all study materials were approved by the Institutional Review Board at the Boston University School of Public Health.

### Manganese in residential tap water samples

**Fig. 1:**
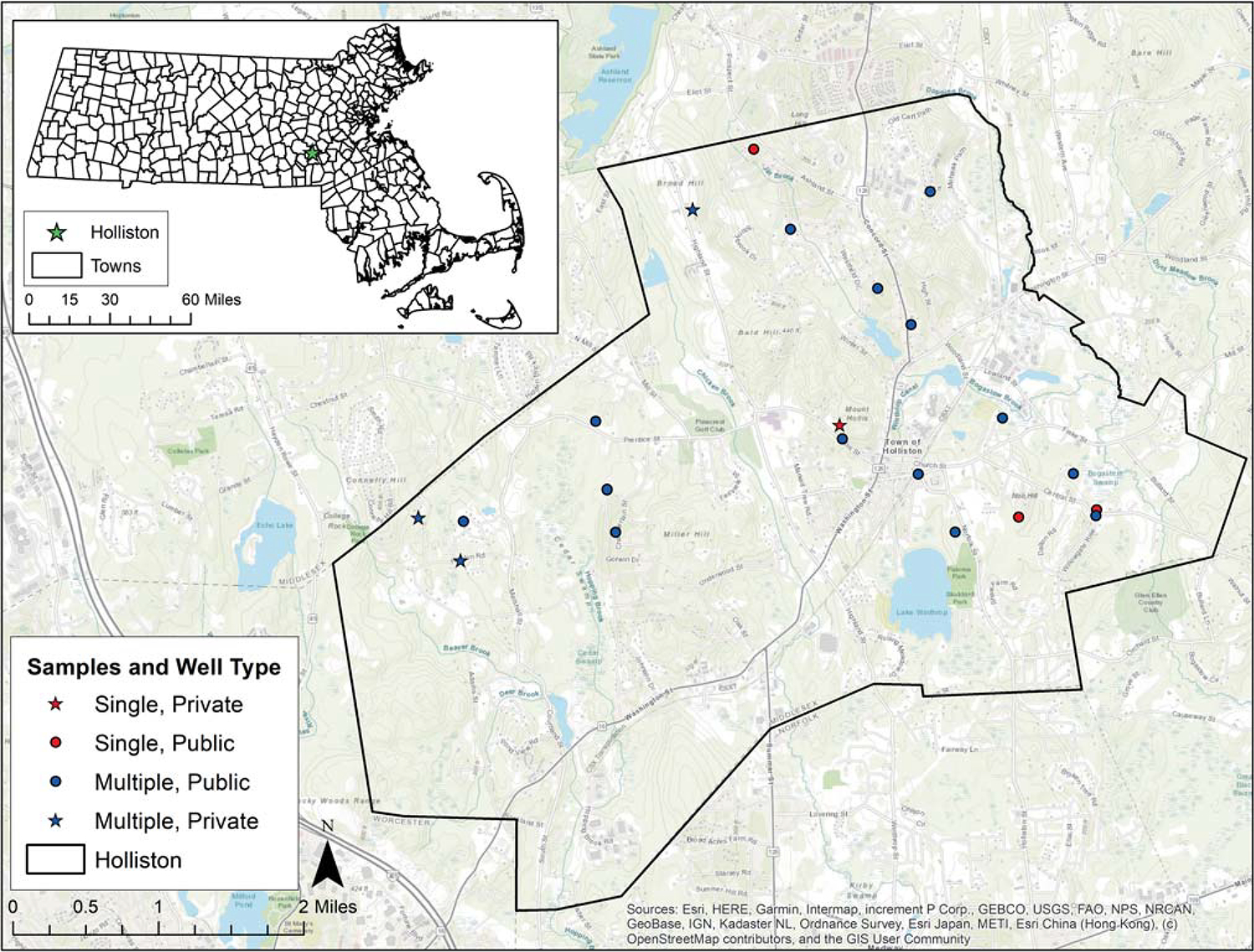
Residential tap water sampling locations from the ACHIEVE study in Holliston, MA, USA between 2018 and 2019. Points represent geocoded jittered participant homes. Stars indicate reliance on private well (vs. public water supply). Red dots and red stars indicate households where only one sample was collected. Blue dots and blue stars indicate households where repeated samples were collected.

Convenience samples of residential tap water were collected by trained study staff every few months between September 2018 and December 2019. At least one sample was collected from each of the 21 households in Holliston, and repeated sampling over nine independent sampling rounds occurred in most (18 of 21) households, with a range of two to nine samples per home. Sampling locations are shown in Fig. 1; dates and number of samples per round are in Supplementary Table 1.

Residential tap water samples were collected using a standardized sampling procedure adapted from the EPA Lead and Copper Rule Compliance Sampling Procedure [26]. Briefly, sample collection occurred as follows: 1) open faucet and allow water to run for 3 min, 2) adjust flow to prevent splashing, 3) rinse the inside of collection tube and cap 3 times with tap water, and 4) collect ~35 mL of water without contacting mouth of tube to faucet. Tap water samples were collected with Easy Reader conical centrifuge tubes (sampling round 1) or VMR metal-free centrifuge tubes (sampling rounds 2 through 9). Samples were taken preferentially from bathtubs or bathroom sinks to obtain unfiltered samples and avoid point-of-use (POU) filtration devices that occur most frequently on kitchen faucets. When unavailable, samples were taken from kitchen faucets free of POU filters and/or with household point of entry (POE) filters turned off. For one household, samples were collected from a sink in the laundry room.

Samples were kept on ice or refrigerated (at 4 °C) and shipped overnight on ice to the University of Illinois Chicago, where they were aliquoted into samples of 10 mL, and acidified with 70% nitric acid (Trace Metal Grade) to reach a pH < 2 in compliance with EPA protocol [27, 28]. Samples were then sent on ice to Northwestern University Quantitative Bio-element Imaging Center to be analyzed for Mn content using inductively coupled plasma-mass spectrometry (ICP-MS) [27]. ICP-MS was performed on a computer-controlled (QTEGRA software) Thermo iCapQ ICP-MS (Thermo Fisher Scientific, Waltham, MA, USA) operating in KED mode and equipped with a ESI SC-2DX PrepFAST autosampler (Omaha, NE, USA). An internal standard was added inline using the prepFAST system and consisted of 1 ng/mL of a mixed element solution containing Bi, In, ^6^Li, Sc, Tb, Y (IV-ICPMS-71D from Inorganic Ventures). Online dilution was carried out by the prepFAST system and used to generate calibration curves consisting of 200, 100, 50, 20, 10, 5, 2, 1 μg/L Mn. The isotopes selected for analysis were ^55^Mn and ^89^Y, ^115^In (chosen as internal standards for data interpolation and machine stability). Instrument performance is optimized daily through autotuning followed by verification via a performance report (passing manufacturer specifications). Additional quality assurance and control measures included collection blinded duplicate samples to provide checks on variability and precision of sampling. One sample was omitted from analyses due to suspected laboratory contamination (2304.2 μg/L vs. other samples from the same household, median [range] = 2.3 μg/L [1.5–3.8 μg/L]). The final analytic sample included 78 tap water samples from 21 households. The average limit of detection (LOD) was calculated by the operating software (Qtegra) based on the calibration curve analyzed at Northwestern. The LOD for water Mn analysis was <0.005 μg/L; all samples were above the LOD [28].

### Publicly available drinking water data

To put case study findings in context with state monitoring data and to provide a historical perspective, we downloaded publicly available data on Mn in well water samples from the Massachusetts Executive Office of Energy and Environmental Affairs (EEA) Drinking Water database (https://eeaonline.eea.state.ma.us/Portal/#!/search/drinking-water). The EEA database contains data on groundwater-based public water supplies (PWS) that source drinking water to at least 25 people for at least 60 days annually. At least one sample from all PWSs in MA are required to be taken annually, though frequency of sample collection can vary based on prior results or specific concerns about contamination [29]. Mn concentrations were measured using EPA methods to measure trace metals in drinking water: EPA 200.7, EPA 200.8, SM 3111B, SM 3113B, or SM 3120B method. We downloaded all available water Mn data in MA. Data were available starting in 1994 and were included for sampling through 2022 in order to have complete years. Between 1994 and 2022, a total of 50,372 water samples were collected and available on the EEA database. We excluded data from raw water samples (i.e., samples prior to any treatment for drinking; *N* = 13,122) to 1) facilitate comparability between our case study and the publicly available data and 2) investigate concentrations in drinking water samples that are reflective of water that is consumed. We removed samples for which LODs were not published (*n* = 40) and replaced data points that were below the LOD (*N* = 12,168; 33%) with LOD/sqrt 2. The final analytical dataset from EEA included 37,181 samples from 320 of 351 MA towns (91%). We also created a separate subset of the data, including only samples collected during the same period as the Holliston case study (September 1, 2018 to December 31, 2019). This subset included 3,642 finished water samples from 252 MA towns (72% of all towns in MA).

### Statistical analysis and geocoding

We calculated summary statistics for tap water Mn concentrations across all participating Holliston households apart of the ACHIEVE study, as well as across repeated samples within households. To assess variability over time by household, we calculated intraclass correlation coefficients (ICC) from linear mixed models. We interpreted an ICC of less than 0.5, which represents poor reliability [30], as indicative of high variability between samples taken from the same home (Holliston ACHIEVE data) or from the same PWS (EEA data). We interpreted an ICC of 0.9 or greater, which represents excellent reliability, as indicative of low variability between samples, and an ICC between 0.50 and 0.90, which represents moderate-to-good reliability, as having moderate variability. Sampling locations in Holliston were geocoded from participant addresses (street number and name) (Fig. 1).

We calculated summary statistics for EEA data across all of MA (i.e., including all towns with available data) and then for Holliston only, and calculated ICCs to assess variability over time by PWS ID. Samples collected back-to-back from the same sampling location on the same day may be considered to be non-independent and including them as independent samples could falsely inflate the percentage of samples exceeding guideline values. To evaluate this, as a sensitivity analysis, we averaged concentrations from samples in the EEA that were taken from the same location on the same date (i.e., “back-to-back” samples; ~4% of samples). Data points were geocoded by town name (EEA data), latitude and longitude (USGS data; Supplementary Fig. 1), and census data (Supplementary Fig. 2). Sources for publicly available data and for generating maps are listed in Supplementary Table 2. All descriptive and statistical analyses were conducted using R 3.5.2 and SAS 9.4 (SAS Institute Inc.). Maps were made using Arc Map 10.7.1.

## RESULTS

### Characteristics of holliston participants

The majority of participants were non-Hispanic white (91%) and had lived in the same home in Holliston since their index child was born (Table 1). Most participating households obtained their drinking water from a public drinking water supply (81%) and used some type of water filtration device (71%). Among participants who reported using a water filtration device, most (60%) used a point-of-use filter, such as a Brita filter or other filter installed on a fixture. The majority of mothers reported that they primarily used either filtered water and/or bottled water for food and beverage (coffee/tea) preparation at time of enrollment. Participants who were available for water sampling (*n* = 21) were similar on most demographic characteristics to ACHIEVE participants who were unavailable for water sampling (*n* = 9) (Supplementary Table 3).

### Manganese in residential tap water samples

**Fig. 2:**
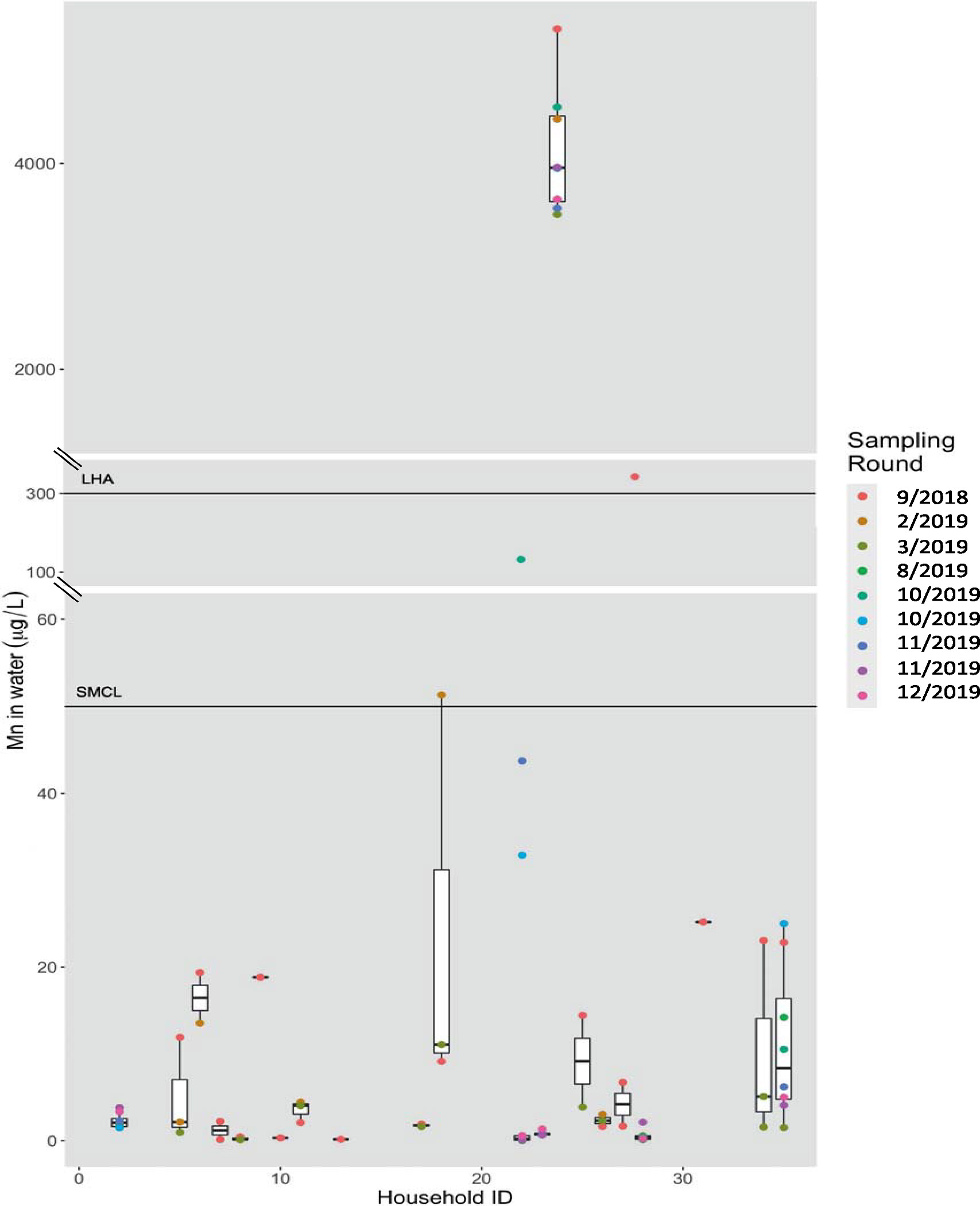
Distribution of water Mn concentrations (μg/L) by ACHIEVE study household (21 households, *n* = 78 samples). Colors represent the sampling round in which samples were collected; month/year of sampling round is indicated in the legend. The horizontal black lines represent the Secondary Maximum Contaminant Level (SMCL) of 50 μg/L and the Lifetime Health Advisory (LHA) of 300 μg/L. Axis is broken between 60 and 100 μg/L, and between 350 and 1000 μg/L, to show the complete range of values.

The median (25th, 75th percentiles) Mn concentration in residential tap water samples was 2.3 μg/L (0.7, 16.6 μg/L) with a range from 0.03 μg/L to 5301.8 μg/L (Table 2). Water Mn concentrations differed by drinking water supply type (median, public water supply = 2.0 μg/L vs. private well = 3535.3 μg/L), although only 14 samples (18%; from 4 households) were collected from homes with private wells. The concentrations of Mn in tap water varied both temporally (i.e., within households) and spatially (i.e., between households) (Fig. 2). Repeated samples of Mn concentrations from the same household were generally similar between sampling rounds (ICC = 0.94); however, for 13 of 18 homes with repeated samples, concentrations in repeated samples collected over time varied at least two-fold. Approximately 14% of samples (from 4 households) exceeded the MA SMCL of 50 μg/L and 12% of samples (from 2 households) were above the MA LHA of 300 μg/L (Table 2; Fig. 2).

### Comparison with publicly available data for Holliston

We compared water Mn concentrations from our case study in Holliston to publicly available data in Holliston during the same sampling period (September 2018 through December 2019). Mn concentrations in our case study were lower compared to samples from the MA EEA database: the median water Mn concentration in our case study 2.3 μg/L (25th, 75th percentile: 0.7, 14.4 μg/L) compared to 11.2 μg/L (3.6, 22.2 μg/L) in EEA monitoring data. However, the range of Mn concentrations in Holliston was greater in our case study (0.03 to 5301.8 μg/L) compared to EEA monitoring data (1.4–147 μg/L) (Table 2). The percent of samples exceeding the SMCL of 50 μg/L in our case study (14%, *N* = 11) was similar to the EEA data (17%, *N* = 3). In contrast, the percent of samples exceeding the LHA of 300 μg/L was higher in our case study (12%, *n* = 9) than in the EEA monitoring data (0%). Notably, nearly all (8 of 9) samples in our study above 300 μg/L were collected from one household with a private well.

**Fig. 3:**
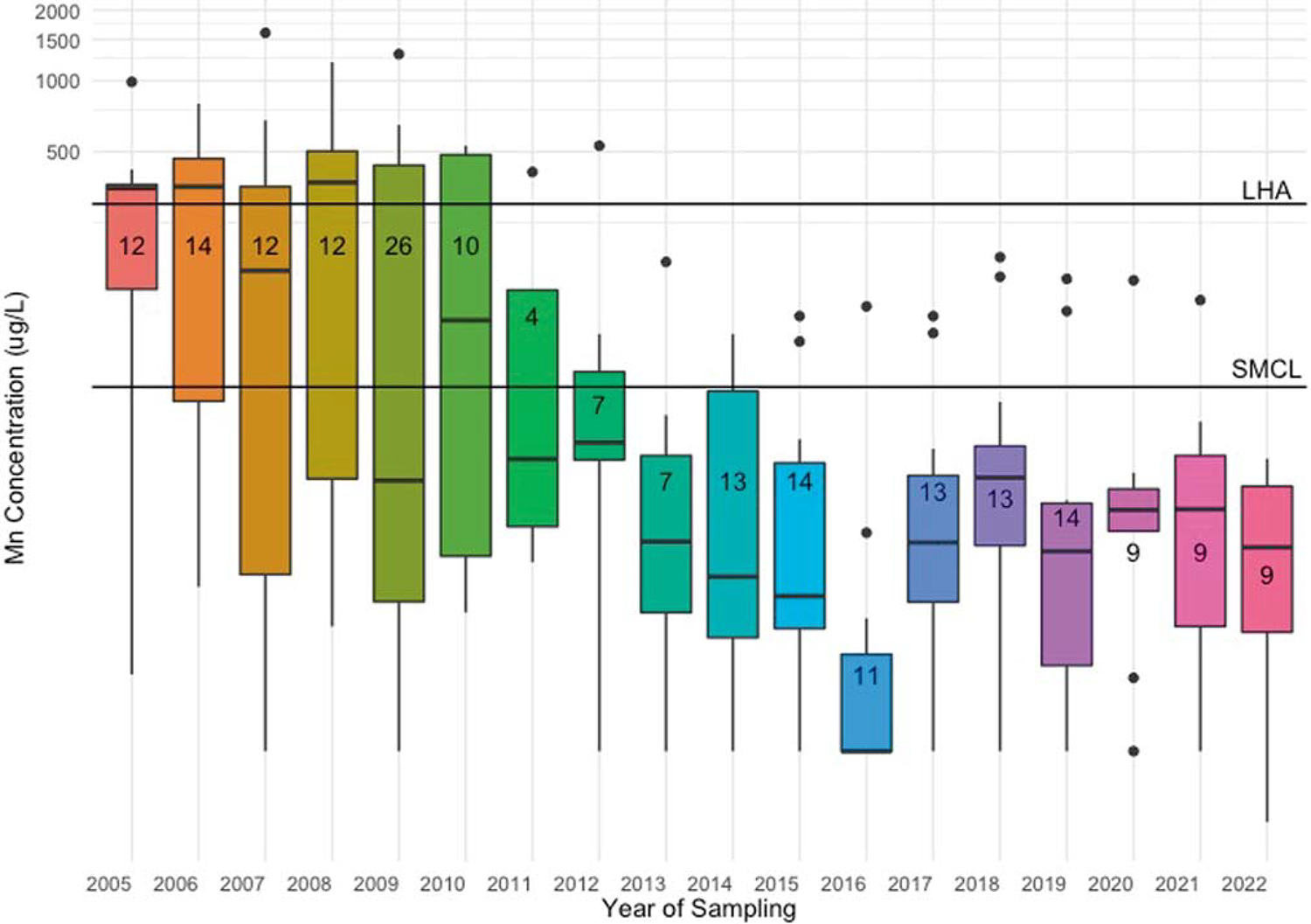
Box plots of manganese (Mn) concentrations in drinking water (μg/L) in Holliston from EEA data between 2005 and 2022. Colored boxes (boxplots) represent 25th to 75th percentiles (interquartile range; IQR) of the distributions; thick black horizontal lines within boxes represent median; vertical lines extend from lowest to largest values (no greater than 1.5 * IQR in either direction); outliers are indicated by black dots (lower outliers: < 1.5 * IQR); higher outliers: > 1.5 * IQR). Numbers on boxplots represent the number of samples collected in given year. SMCL Secondary maximum contaminant level (50 μg/L). LHA=lifetime health advisory (300 μg/L). Note: Y-axis is on logarithmic scale.

We then evaluated temporal variability in Mn water concentrations for Holliston by examining all available data from the EEA database from 2005 to 2022 (Fig. 3). A total of 209 water samples were collected (equivalent to an average of 12 samples per year, from one PWS in Holliston). The median Mn concentration was 15.4 μg/L (25th, 75th percentile: 5.5, 117.0 μg/L) and concentrations ranged from 0.7 to 1600 μg/L across sampling years (Table 2). More temporal variability was evident during this longer sampling period (ICC = 0.32) compared to the time frame of our (2018–2019) case study (ICC = 0.94). Between 2005 and 2022, 33% of samples in Holliston exceeded the SMCL and 19% exceeded the LHA. Since 2005, water Mn concentrations in Holliston have declined and become less variable (Fig. 3). However, several samples in more recent years still exceeded aesthetic and health-based guidelines.

### Spatial and temporal variability of publicly available data for MA

**Fig. 4:**
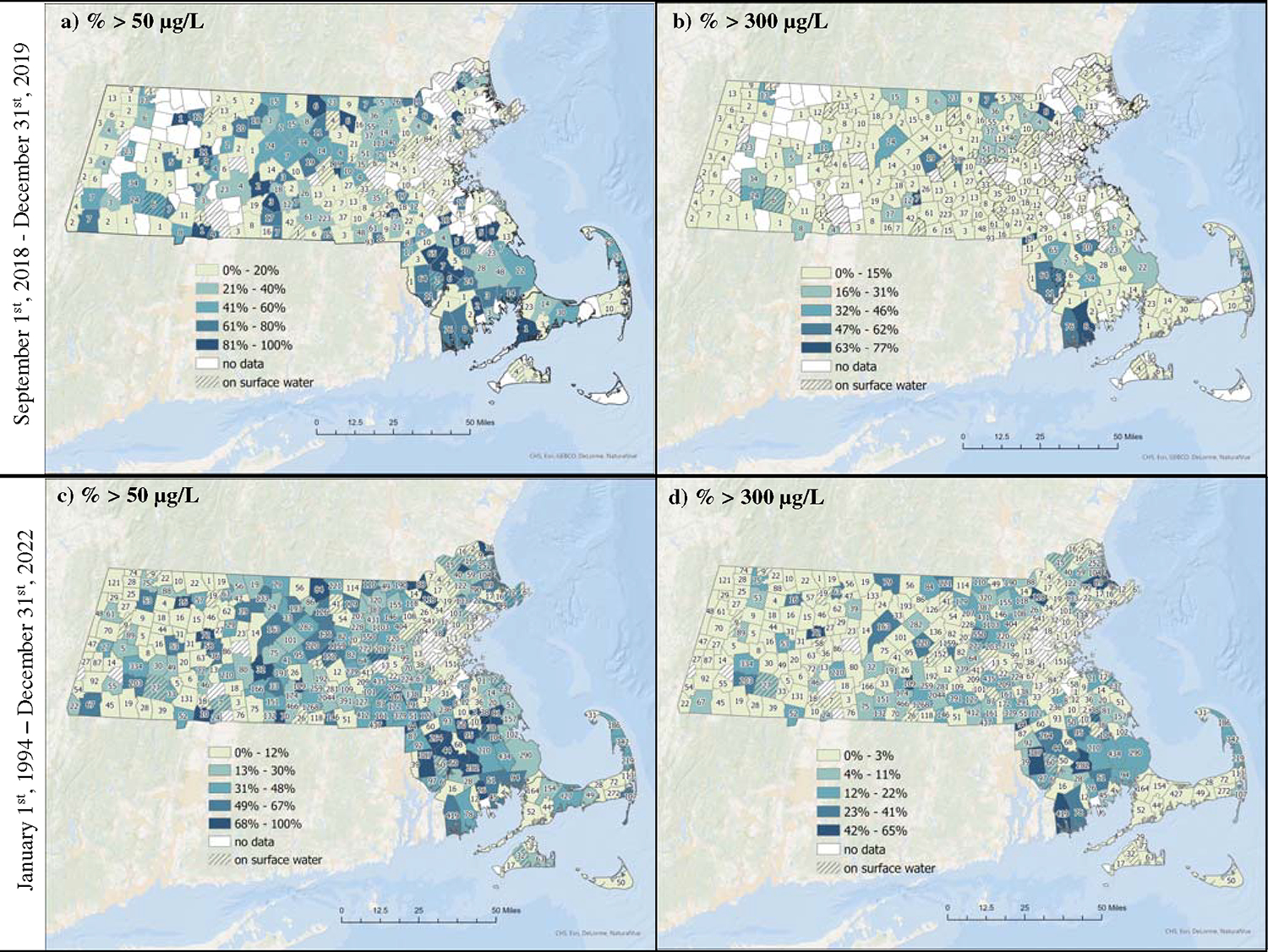
Percent of samples from EEA database in Massachusetts exceeding the secondary maximum contaminant level (SMCL) of 50 μg/L or lifetime health advisory (LHA) of 300 μg/L. Darker shading represents greater percentage of samples above guideline values. Number of samples in each town is labeled in text within town lines. **a** Percent of samples collected between September 2018 and December 2019 above the SMCL. **b** Percent of samples collected between September 2018-December 2019 above the LHA. **c** Percent of samples collected between 1994 and 2022 above the SMCL. **d** Percent of samples collected between 1994 and 2022 above the LHA.

The statewide median water Mn concentration during the case study period (September 2018–December 2019) was 17.0 μg/L (25th, 75th percentile: 3.5, 135.0 μg/L), which is higher than in Holliston alone (median, case study: 2.3 μg/L; EEA data: 11.2 μg/L) (Table 2). There was a large range of Mn concentrations measured in drinking water samples (range: 1–17,000 μg/L) from statewide EEA data, which was similar to our case study. Mn concentrations were above the SMCL in more than one third (37%, *n* = 1344) of all samples, and above the LHA in 13% (*n* = 484). Fewer towns in the northwestern part of MA were represented in the EEA database during this time frame, which suggests less frequent sampling in that part of the state (Fig. 4a, b). Towns in southern MA had the highest percentage of samples exceeding guideline values (Fig. 4a, b). Over half (62%; *N* = 157) of towns with available data experienced at least one sample above the SMCL of 50 μg/L and 28% (*N* = 71) experienced at least one sample above the LHA of 300 μg/L. In contrast to the EEA data in Holliston alone, concentrations from repeated sampling locations (i.e., PWSs) across the state between September 2018 and December 2019 had greater temporal variability (ICC = 0.43). This may be due in part to a larger number of repeated samples (median # of samples per town: 16; range: 2–176), the presence of outlier values in some PWSs, and/or intrinsic variabilities in Mn levels driven by hydrogeochemical dynamics.

Lastly, we examined descriptive statistics and spatial trends of all publicly available data in MA (Table 2; Fig. 4c, d). Between 1994 and 2022, 37,181 samples from 91% (*N* = 320) of all towns in MA were collected. The median water Mn concentration was 17.0 μg/L and ranged from 0.7 to 159,000 μg/L. Over the 29 years of sampling, 35% (n = 13,035) of samples exceeded the SMCL and 12% (*n* = 4328) exceeded the LHA. Most towns with available data in the EEA database had at least one sample above the SMCL (77%; *N* = 247) and 49% (*N* = 156) of towns had at least one sample above the LHA during this time frame. The ICC was 0.35, which suggests high temporal variability (Table 2) in addition to the spatial variability observed across the state (Fig. 4c, d). We did not observe a clear spatial pattern, though the majority of towns with the highest percentage of samples above guideline values were in southern MA (Fig. 4c, d). Across MA, the number of samples collected between 1994 and 2022 ranged from 1–4,433 samples per year, with fewer samples collected prior to 2005 (Supplementary Table 4). This may reflect less frequent monitoring of Mn during this time and/or incomplete data in the EEA system. While the annual median Mn concentrations in MA have decreased over time, high concentrations of Mn occur regularly with an average of 42% of samples exceeding the SMCL of 50 μg/L and 9% exceeding the LHA of 300 μg/L each year. In our sensitivity analysis where we averaged back-to-back samples, descriptive statistics and percentage of exceedances overall were identical (Supplementary Table 5).

## DISCUSSION

In this case study of a suburban MA community, average concentrations of Mn in residential tap water (median = 2.3 μg/L) were generally lower than U.S guideline values. However, there was a wide range of water Mn concentrations (up to 5300 μg/L) and about 12–14% of samples exceeded U.S guidelines for Mn concentrations in drinking water. In addition to temporal variability within households, there was spatial variability in our Holliston case study where concentrations varied (up to 1000-fold) between participant homes (Fig. 2). Across the state of MA, Mn water concentrations were highly variable from 1994 to 2022, and although median concentrations of Mn in drinking water in Holliston and across the state of MA have declined since the early 2000s, we observed a consistent pattern where about one-third of samples exceed the SMCL and 10–15% exceed the LHA annually (Supplementary Table 4). Spatial variability in Mn concentrations across MA is also evident, with towns in central and southern MA having larger proportions of samples with concentrations above aesthetic and health-based guidelines compared to other parts of the state (Fig. 4). Approximately 2 million people reside in these impacted regions across MA; in most of these towns, a high proportion of residents rely on groundwater as their main source of drinking water (Supplementary Fig. 2). Thus, this study demonstrates large spatial and temporal variability in water Mn concentrations in MA, and given the limited sampling frequencies for monitoring data, more frequent sampling is needed to fully characterize Mn in drinking water.

While Mn is an essential nutrient that is required for healthy growth and development, excess Mn has been associated with adverse neurodevelopmental outcomes in children [2, 11, 31]. The U.S lifetime health advisory for Mn in drinking water was derived based on consumption for an average healthy adult and may not be protective for more susceptible populations like children [6]. As such, MA has developed a 10-day LHA of 300 μg/L for children under 1 year old, inferring that infants should not consume drinking water with Mn concentrations above 300 μg/L for more than 10 days in a year [29]. As we observed in this study, concentrations within Holliston and across the state of MA exceed 300 μg/L, thereby placing infants at risk of elevated Mn exposure and associated health effects. In addition, given the historical and regular contamination of formula and infant foods with heavy metals [16, 32, 33], and the supplementation of certain foods (e.g., infant formula) with Mn, it is plausible that, when water containing high concentrations of Mn is used to make formula, infants may be receiving a “double dose” of Mn that could result in excess exposure [16]. Given the potential for exposure to Mn from multiple sources, a protective drinking water standard for Mn warrants consideration [34].

The World Health Organization (WHO) publishes non-enforceable guidelines for drinking water that are intended to promote the protection of public health by advocating for adoption of proposed guidelines as enforceable standards [35]. In 2011, the World Health Organization (WHO) proposed a drinking water guideline concentration of 400 μg/L [35]. However, the WHO did not adopt this guideline because they considered 400 μg/L to be “well above the concentrations of Mn typically found in drinking water”, and as such, it was “not necessary to derive a formal guideline value” [35, 36]. Evidence from our case study and from publicly available MA EEA data, as well as data from the USGS report^3^, U.S. UCMR [9], and studies in Bangladesh [19, 37–40], Pakistan [41, 42], India [43], Canada [44–46] and Brazil [47] demonstrate that Mn concentrations in drinking water routinely exceed 400 μg/L. More recently, the WHO published an update to the previous guideline of 400 μg/L and determined a provisional health-based guideline value of 80 μg/L based on health considerations for bottle-fed infants [48]. This provisional health-based guideline incorporates more recent epidemiological studies of water Mn and neurodevelopmental outcomes in children. However, this guideline is non-enforceable, remains provisional, and may not be protective of the most sensitive populations. While Mn was a candidate on the UCMR third (2012–2015) and fourth (2018–2020) programs, Mn is not included in the most current UCMR sampling list (UCMR 5; 2023–2025), suggesting there may be even less federal monitoring of water Mn in the coming years [9].

Prior epidemiology studies have reported associations between water Mn and adverse neurodevelopmental outcomes in children. In two Canadian studies, where concentrations of water Mn ranged between 1 and 2700 μg/L, water Mn was associated with altered motor function, problems with classroom behavior, and hyperactivity in school-age children [18, 22]. Studies in Bangladesh, where average drinking water Mn concentrations are higher than in most parts of North America (range: ~1–8600 μg/L), have reported adverse associations between water Mn (mean: 795 μg/L) and IQ [19] as well as academic performance (mean: 889 μg/L; median 651 μg/L) [20]. In a large nationwide study of 643,401 Danish children, where most water Mn concentrations were lower than in other studies (<100 μg/L; over 80,000 samples measured across 15 years), authors reported that children who were exposed to higher levels of Mn in drinking water had a higher risk of certain subtypes of attention-deficit hyperactivity disorder [49]. In a U.S.-based semi-ecologic investigation in North Carolina, authors reported a positive association between aggregate mean county-scale water Mn concentrations in private wells and prevalence of delayed milestones and hearing loss in children 0–35 months old [50]. County-scale water Mn concentrations in private wells in this study ranged from 15 to 1116 μg/L (mean of census track) and 21% of samples exceeded the SMCL of 50 μg/L. Overall, these findings suggest that Mn in drinking water, even at levels lower than current guideline values, are associated with adverse health outcomes for children. While most of the pediatric epidemiological literature to date on water Mn and neurodevelopment has been conducted outside the U.S., the range of water Mn concentrations in the aforementioned studies are within the ranges measured in our Holliston case study, in the publicly available EEA data, and in USGS and UCMR data, reported by McMahon et al., 2019 [3] and Eaton, 2021 [9], respectively. Collectively, the available data suggest that Mn is present in U.S. drinking water at levels that can adversely impact children’s health, which warrants public health attention.

In our comparison with publicly available data, we examined Mn levels in finished water samples only. As expected, Mn concentrations in raw samples were higher than in finished water samples (between 1994 and 2022, median: 110 μg/L in raw, 17 μg/L in finished samples). Consistent with the differences in distributions, the percent of samples above SMCL and LHA for raw samples was higher: 61% above the SMCL and 33% above the LHA, compared to 35% and 12% in finished samples. This is expected given that the role of processing water is in part to reduce the levels of chemical, physical and biological contaminants. Finished samples may still contain levels of Mn and/or other contaminants due to differences in processing between facilities, contamination after processing from either natural or anthropogenic sources and/or differences in infrastructure. Notably, Mn will not be effectively removed from water through the use of most point of use filters, and the most effective methods for removing Mn (e.g., reverse osmosis) is costly and/or burdensome to maintain for consumers [6, 46]. Further, in the U.S. and globally, a large proportion of the population consuming groundwater uses private or community wells/boreholes without any treatment. As such, upstream solutions to reducing the levels of Mn in drinking water to protect health are warranted.

As a case study, we had a small sample size and thus limited statistical power. However, we collected up to nine repeated tap water samples per household, which increased sample size and allowed us to explore temporal variability; this has not been documented widely within the U.S or globally. Further, our findings of spatial and temporal variability are consistent with a similar study in Canada and with what we observed in publicly available monitoring data from MA [46]. We also utilized publicly available data to place our findings into context with state levels and to describe temporal and spatial patterns in water Mn concentrations. The observed temporal and spatial variability could be due to differences in geographical and hydrological features between sample locations, season of individual samples, and/or other weather characteristics. However, data were not available for every MA town and were especially limited for towns that utilize surface water as a drinking water source; this illustrates the poor resolution in water sampling (Fig. 3; Supplementary Fig. 2). Further, we do not have access to information about reasons for sampling frequency and/or if there was missing data in the EEA database. Though, we explored whether or not sample quantity within in a town was correlated with the percent of exceedances of the SMCL and LHA and found these were weakly correlated (r for %SMCL: 0.22; for %LHA: 0.28). Lastly, we were unable to characterize Mn concentrations in private wells (outside of our case study), which may be more vulnerable to higher levels of Mn and other geogenic co-contaminants because they lack water treatment [3, 51].

Despite limitations, our study nonetheless provides an examination of repeated concentrations of Mn in residential drinking water, data which are particularly rare in the U.S. We also put case study findings into context with publicly available data and report on trends for the state of MA. While some studies reported on Mn water concentrations in the U.S., these studies were limited to small number of sampling locations and to a limited number of repeated samples. Prior work using USGS samples included 1,023 samples (from 310 wells in 75 towns) between 1998 and 2014 with an unequal distribution of samples across towns (Supplementary Fig. 1). For example, for three towns on Cape Cod, there were between 27 and 113 samples collected, whereas for most other MA towns, only 1–7 samples were collected over 16 years, with most repeat samples being collected hours apart on the same day. Reasons for sampling frequency were not available but may be related to the presence of other co-contaminants or to other drinking water concerns.

Although our case study relied on a convenience sample of participants who were available during sampling visits, participants who were available for sampling were similar to those who were not available with regards to most characteristics such as maternal race/ethnicity, public versus private well use, and current drinking water consumption patterns (Supplementary Table 3). We also varied the day and time for sampling to capture as many different households as possible and in order to minimize the influence of employment, childcare or other barriers on sample availability as well as to maximize spatial representation throughout Holliston. This case study is part of a community-initiated pilot study; as such, we worked closely with community members throughout the study, which helped to increase study participation and retention as well as to facilitate report-back of study results.

The safe level of Mn concentrations in drinking water is unknown. Evidence from pediatric epidemiological literature suggests that Mn in drinking water may be associated with adverse neurodevelopmental effects. We observed that concentrations of water Mn vary temporally and spatially in MA, based on data from a small suburban town as well as from statewide monitoring data. Given evidence that concentrations of Mn in drinking water regularly exceed both aesthetic and health-based guidelines, more temporally- and spatially-resolved individuallevel drinking water data are needed. Research identifying predictors of Mn concentrations in drinking water and examining links between drinking water Mn concentrations and children’s health are warranted.

## Supplementary Material

Supplementary Material

## Figures and Tables

**Table 1.** Demographic information, drinking water characteristics and consumption patterns among Holliston ACHIEVE case study participants (*n* = 21).

	*N (%)*
**Maternal race/ethnicity**	
Non-Hispanic White	19 (91%)
Multicultural/Other	2 (9%)
**Moved homes within Holliston since childbirth**	3 (14%)
**Household water source**	
Public water supply	17 (81%)
Private well	4 (19%)
**Has water filtration system in home**	15 (71%)
*Point-of-entry (basement)*	4 (26%)
*Point-of-use (sink, pitcher)*	9 (60%)
*Other*	2 (13%)
**Use of filtered water for food preparation**	
Always	5 (24%)
Rarely/Sometimes	7 (33%)
Never	9 (43%)
**Use of filtered water for coffee/tea preparation**	
Always	12 (57%)
Rarely/Sometimes	4 (19%)
Never	5 (24%)
**Bottled water use**	
Always	8 (38%)
Rarely/Sometimes	9 (43%)
Never	4 (19%)

**Table 2.** Descriptive statistics of manganese concentrations for water samples collected from ACHIEVE case study and from publicly available EEA database.

	*N*	LOD (μg/L)	*N* (%) > LOD	Min	Median	Mean	Max	GM (95% CI)	ICC^a^	I (%) > 50 μg/L^b^	*N* (%) > 300 μg/L^c^
**Holliston**											
ACHIEVE pilot study 9/2018–12/2019	78	<0.005	78 (100)	0.03	2.3	432.9	5,301.8	4.6 (2.4, 9.0)	0.94	11 (14)	9 (12)
EEA 9/2018–12/2019	20	2.0	17 (90)	1.4	11.2	29.8	147	11.3 (5.8, 22.3)	0.94	3 (17)	0 (0)
EEA 2005–2022	209	0.3–50	179 (86)	0.7	15.4	132.7	1,600	23.8 (18.2, 31.2)	0.32	68 (33)	40 (19)
**All of MA**											
EEA 9/2018–12/2019	3,642	0.1–50	2,459 (68)	0.1	17.0	154.8	17,000	23.4 (21.2, 25.0)	0.43	1,344 (37)	484 (13)
EEA 1994–2022	37210	0.1–100	25,020 (67)	0.1	17.0	135.4	159,000	21.5 (21.0, 22.0)	0.35	13,035 (35)	4,328 (12)

*LOD* Limit of Detection, *GM* Geometric mean, *CI* Confidence interval.

a Calculated from a linear mixed model where household ID (ACHIEVE) or PWS ID (EEA) was set as random intercept (i.e., indicator of repeated samples).

b 50 μg/L: Secondary maximum contaminant level set by US EPA and MA Department of Environmental Protection [6].

c 300 μg/L: Lifetime Health Advisory set by US EPA; [6] 10-day health advisory for children under 1 year old MA [29].

## Data Availability

The datasets generated during and/or analyzed during as part of the case study are not publicly available due privacy concerns. The publicly datasets analyzed from the Massachusetts Energy and Environmental Affairs database are available at their website (https://eeaonline.eea.state.ma.us/Portal/#!/search/drinking-water) and links to all sources used in map generation are included in Supplementary Table 2.

## IMPACT STATEMENT:

- This study is one of the first to examine Mn concentrations in drinking water both spatially and temporally in the U.S. Findings suggest that concentrations of Mn in drinking water frequently exceed current guidelines and occur at concentrations shown to be associated with adverse health outcomes, especially for vulnerable and susceptible subpopulations like children. Future studies that comprehensively examine exposure to Mn in drinking water and its associations with children’s health are needed to protect public health.
