# Supplementary material for: Manganese in residential drinking water from a community-initiated case study in Massachusetts
Source: J Expo Sci Environ Epidemiol. Author manuscript; Available in PMC 2024 Mar 14. (PMC10727146; doi:10.1038/s41370-023-00563-9)
Supplement: Supplementary Material [file NIHMS1948933-supplement-Supplementary_Material.docx]

**Supplemental Material**

Manganese Concentrations in Residential Tap Water Samples from a Community-Initiated Case Study in Massachusetts:

Alexa Friedman^1^, Elena Boselli^2^, Yelena Ogneva-Himmelberger^3^, Wendy Heiger-Bernays^1^, Paige Brochu^1^, Mayah Burgess^1^, Samantha Schildroth^1^, Allegra Denehy^4^,Timothy Downs^3^, Ian Papautsky^2^, Birgit Claus Henn^1^

Affiliations:

1. Department of Environmental Health, Boston University School of Public Health, Boston, Massachusetts, USA
2. Department of Biomedical Engineering, University of Illinois Chicago, Chicago, Illinois, USA
3. Department of International Development, Community, and Environment, Clark University, Worcester, Massachusetts, USA
4. Holliston Community Member, Holliston, Massachusetts, USA

Number of Figures:2

Number of Tables: 5

**Supplemental Figure 1.** United States Geographical Survey Data for Massachusetts between 1998-2014 downloaded from McMahon et al., 2019.

**Supplemental Figure 2.** Population sizes and percent of towns served by ground water in MA.

**Supplemental Table 1.** Dates of sampling rounds and number of samples per round

**Supplemental Table 2.** Publicly available data and geodatabase sources

**Supplemental Table 3.** Demographic information , drinking water characteristics and consumption patterns among all ACHIEVE participants (n=30)

**Supplemental Table 4.** Distribution of water Mn concentrations from EEA data between 1994-2022

**Supplemental Table 5.** Descriptive statistics of manganese concentrations for water samples collected from EEA database comparing results when averaging back-to-back samples

**Supplemental Figure 1. United States Geographical Survey Data for Massachusetts between 1998-2014.** Data was downloaded from McMahon et al., 2019,^[[1]](#footnote-1)^ which includes 1,023 samples from 310 individual wells located in 75 towns in MA. For each well there were several samples available, so we calculated the mean manganese concentration in each well. The number of samples from each town are labeled in text. Each sample was labeled with a and longitude and data was geocoded and summarized by town to calculate percent wells where the mean Mn concentration was above 50 µg/L (panel a) or and 300 µg/L (panel b).

**Supplemental Figure 2. Population sizes and percent of towns served by ground water in MA.**

| **Supplemental Table 1.** Dates of sampling rounds and number of samples per round | | |
| --- | --- | --- |
| Sampling Round | Date(s) | Number of Samples |
| 1 | 09/10/2018 – 09/12/2018 | 22 |
| 2 | 02/26/2019 – 02/27/2019 | 9 |
| 3 | 03/19/2019 – 03/21-2019 | 14 |
| 4 | 08/20/2019 | 5 |
| 5 | 10/08/2019 – 10/09/2019 | 7 |
| 6 | 10/29/2019 | 5 |
| 7 | 11/05/2019 | 5 |
| 8 | 11/18/2019 - 11/20/2019 | 6 |
| 9 | 12/10/2019 – 12/11/2019 | 6 |

| **Supplemental Table 2. Publicly available data and geodatabase sources** | | |
| --- | --- | --- |
| **Data Layer** | **Source** | **Date Downloaded** |
| Massachusetts Town Boundary | MassGIS: https://www.mass.gov/info-details/massgis-data-2020-us-census-towns | January 23^rd^, 2023 |
| Population served by ground water | EPA’s Consumer Confidence Reports (CCR) for Massachusetts - https://ordspub.epa.gov/ords/safewater/f?p=136:103::::RP,103:P103_STATE:MA  For town | January 23^rd^, 2023 |
| Town population | 2020 Census data – UMASS Donahue Institute: https://donahue.umass.edu/business-groups/economic-public-policy-research/massachusetts-population-estimates-program/census-2020-data-for-massachusetts | January 23^rd^, 2023 |
| Manganese concentrations | Massachusetts Department of Energy and Environmental Affairs:  https://eeaonline.eea.state.ma.us/portal#!/search/drinking-water/results?ChemicalName=MANGANESE | January 23^rd^, 2023 |
|  | USGS (McMahon et al., 2019): https://pubs.acs.org/doi/suppl/10.1021/acs.est.8b04055/suppl_file/es8b04055_si_002.xlsx | January 23^rd^, 2023 |

| **Supplemental Table 3.** Demographic information , drinking water characteristics and consumption patterns among all ACHIEVE participants (n=30) | | |
| --- | --- | --- |
|  | Participants (N=21)  N(%) | Non-participants* (N=9)  N(%) |
| **Maternal race/ethnicity** |  |  |
| Non-Hispanic white | 19 (91%) | 9 (100%) |
| Multicultural/Other | 2 (9%) |  |
| **Has moved since childbirth**, yes | 3 (14%) | 3 (33%) |
| **Household water source** |  |  |
| Public water supply | 17 (81%) | 8 (89%) |
| Private well | 4 (19%) | 1 (11%) |
| **Has filtration system, Yes** | 15 (71%) | 5 (56%) |
| *Point of entry (basement)* | 4 (27%) | 4 (80%) |
| *Point of use (sink, pitcher)* | 9 (60%) | 1 (20%) |
| *Other* | 2 (13%) | 0 |
| **Use of filtered water for food preparation** |  |  |
| Always | 5 (24%) | 2 (22%) |
| Rarely / Sometimes | 7 (33%) | 2 (22%) |
| Never | 9 (43%) | 4 (44%) |
| Don’t know | 0 | 1 (11%) |
| **Use of filtered water for coffee/tea preparation** |  |  |
| Always | 12 (57%) | 4 (44%) |
| Rarely / Sometimes | 4 (19%) | 2 (22%) |
| Never | 5 (24%) | 2 (22%) |
| Don’t know | 0 | 1 (11%) |
| **Bottled water use** |  |  |
| Never | 4 (19%) | 4 (44%) |
| Rarely / Sometimes | 9 (43%) | 4 (44%) |
| Always | 8 (38%) | 1 (11%) |
| Don’t know | 0 | 0 |
| *Non-participants refer to members of the original ACHIEVE pilot study who were not a part of water sampling in the present case study | | |

| **Supplemental Table 4.** Distribution of water Mn concentrations from EEA data between 1994-2022 | | | | | | | | | |
| --- | --- | --- | --- | --- | --- | --- | --- | --- | --- |
| **Year** | **N** | **# below LOD** | **LOD** | **Minimum** | **Median** | **Mean** | **Maximum** | **#(%) above SMCL** | **#(%) above LHA** |
| 1994 | 1 | 0 | 10 | 70.0 | 70.0 | 70.0 | 70.0 | 1 (100) | 0 (0) |
| 1996 | 1 | 0 | 1 | 76.0 | 76.0 | 76.0 | 76.0 | 1 (100) | 0 (0) |
| 1997 | 3 | 3 | 10 | 7.1 | 7.1 | 7.1 | 7.07 | 0 (0) | 0 (0) |
| 1998 | 1 | 0 | 16 | 150.0 | 150.0 | 150.0 | 150.0 | 1 (100) | 0 (0) |
| 1999 | 2 | 2 | 20 | 14.1 | 14.1 | 14.1 | 14.1 | 0 (0) | 0 (0) |
| 2001 | 1 | 0 | 1 | 32.0 | 32.0 | 32.0 | 32.00 | 0 (0) | 0 (0) |
| 2002 | 23 | 1 | 1-10 | 7.1 | 165.0 | 139.7 | 273.0 | 14 (61) | 0 (0) |
| 2003 | 4 | 1 | 1-50 | 7.1 | 98.5 | 88.5 | 150.0 | 3 (75) | 0 (0) |
| 2004 | 50 | 14 | 1-50 | 0.7 | 70.2 | 317.5 | 3,900.0 | 28 (56) | 12 (24) |
| 2005 | 454 | 90 | 1-50 | 0.7 | 45.8 | 146.5 | 1,800.0 | 220 (48) | 67 (15) |
| 2006 | 607 | 170 | 1-50 | 0.7 | 38.0 | 164.4 | 14,000.0 | 272 (45) | 77 (13) |
| 2007 | 723 | 206 | 0.5 - 50 | 0.4 | 30.0 | 137.9 | 8,150.0 | 303 (42) | 92 (13) |
| 2008 | 943 | 260 | 0.5 - 50 | 1.4 | 26.0 | 158.7 | 3,000.0 | 382 (41) | 138 (15) |
| 2009 | 857 | 266 | 0.5 - 50 | 1.0 | 26.0 | 170.6 | 3,280.0 | 352 (41) | 132 (15) |
| 2010 | 844 | 290 | 0 - 50 | 0.7 | 22.0 | 136.7 | 2,800.0 | 312 (37) | 111 (13) |
| 2011 | 1,375 | 475 | 0 - 50 | 0.7 | 20.0 | 108.7 | 2,200.0 | 458 (33) | 140 (10) |
| 2012 | 1,515 | 625 | 0.1 - 50 | 0.4 | 20.0 | 95.3 | 3,260.0 | 433 (29) | 121 (8) |
| 2013 | 1,670 | 634 | 0.1 - 50 | 0.4 | 17.7 | 98.6 | 8,650.0 | 467 (28) | 150 (9) |
| 2014 | 4,433 | 1491 | 0.1 - 50 | 0.1 | 11.0 | 104.4 | 4,550.0 | 1,358 (31) | 413 (9) |
| 2015 | 3,538 | 925 | 0.1 - 50 | 0.4 | 23.0 | 132.7 | 4,600.0 | 1,406 (40) | 439 (12) |
| 2016 | 3,055 | 844 | 0.1 - 50 | 0.7 | 23.0 | 157.9 | 4,881.0 | 1,239 (41) | 458 (15) |
| 2017 | 3,697 | 1223 | 0.1 - 50 | 0.2 | 14.1 | 171.6 | 159,000.0 | 1,236 (33) | 427 (12) |
| 2018 | 2,814 | 757 | 0.1 - 50 | 0.4 | 25.9 | 166.5 | 17,00000 | 1,141 (41) | 401 (14) |
| 2019 | 2,842 | 988 | 0.1 - 50 | 0.7 | 15.0 | 145.4 | 52,80.0 | 1,004 (35) | 365 (13) |
| 2020 | 3,125 | 1254 | 0.1 - 50 | 0.4 | 11.0 | 99.9 | 5,410.0 | 898 (29) | 266 (9) |
| 2021 | 2,397 | 832 | 0 - 50 | 0.1 | 16.0 | 139.5 | 6,960.0 | 837 (35) | 293 (12) |
| 2022 | 2,206 | 810 | 0 - 50 | 0.1 | 11.0 | 137.0 | 18,000.0 | 669 (30) | 226 (10) |
| N= sample size; LOD = limit of detection; SMCL = secondary maximum contaminant level of 50 µg/L; LHA = lifetime health advisory of 300 µg/L | | | | | | | | | |

| **Supplemental Table 5:** Descriptive statistics of manganese concentrations for water samples collected from EEA database comparing results when averaging back-to-back samples | | | | | | | | | | |
| --- | --- | --- | --- | --- | --- | --- | --- | --- | --- | --- |
|  | N | LOD (µg/L) | N (%) > LOD | Min | Median | Mean | Max | GM  (95% CI) | N (%) > 50 µg/L^a^ | N (%) > 300 µg/L^b^ |
| EEA  1994-2022 | 37,210 | 0.1 – 100 | 25,020 (67) | 0.1 | 17.0 | 135.4 | 159,000 | 21.5  (21.0, 22.0) | 13,035 (35) | 4,328 (12) |
| EEA  1994-2022* | 36,200 | 0.1 – 100 | 24,313 (67%) | 0.1 | 17.0 | 134.1 | 159,000 | 21.2  (20.8, 21.6) | 12,595 (35) | 4,167 (12) |
| LOD: Limit of Detection; GM: geometric mean; CI: confidence interval  a 50 µg/L: Secondary maximum contaminant level set by US EPA and MA Department of Environmental Protection[20]  b 300 µg/L: Lifetime Health Advisory set by US EPA; [20] 10-day health advisory for children under 1 year old MA[21]  *Data were samples on matching public water system, location ID and Date were averaged together | | | | | | | | | | |

1. Peter B. McMahon et al., “Elevated Manganese Concentrations in United States Groundwater, Role of Land Surface–Soil–Aquifer Connections,” *Environmental Science & Technology* 53, no. 1 (January 2, 2019): 29–38, https://doi.org/10.1021/acs.est.8b04055. [↑](#footnote-ref-1)
