# Supplementary material for: Manganese in residential drinking water from a community-initiated case study in Massachusetts
Source: J Expo Sci Environ Epidemiol. 2023 Jun 10;34(1):58–67. doi: 10.1038/s41370-023-00563-9 (PMC10727146; doi:10.1038/s41370-023-00563-9)
Supplement: Supplementary file 1 [file 41370_2023_563_MOESM2_ESM.pdf]

SPRINGER NATURE

Corresponding Author name: Alexa Friedman

Manuscript Number: JESEE-23-4230.R1

Reporting Checklist

This checklist is used to ensure the quality, transparency, and reproducibility of published results. We require authors attest that these components have been considered and addressed.

**Exposure Assessment Guiding Principle**	**Yes/No/Not Applicable**
Has the method to estimate exposure been described clearly?	Yes
Has the exposure assessment method been validated/evaluated as a proxy for exposure and is its validity or agreement with other methods described?	Yes
Is the time period over which the exposure assessment method is considered to be a proxy for exposure appropriate for the research question?	Yes
If exposure is modeled or measured, were all critical potential routes and sources of exposure considered?	Not Applicable
If exposure is modeled, how does it vary over space and time and are necessary historical data incorporated?	Yes
If biomarkers are used as indicators of exposure, could the biomarker measurement have been affected by the outcome (i.e., reverse causality)?	Not Applicable
Are the strengths and weaknesses of the exposure approach detailed and discussed?	Yes
